# Supplementary figures and images for: Development of multiplex real-time PCR for rapid identification and quantitative analysis of Aspergillus species
Source: PLoS One. 2020 Mar 9;15(3):e0229561. doi: 10.1371/journal.pone.0229561 (PMC7062252; doi:10.1371/journal.pone.0229561)

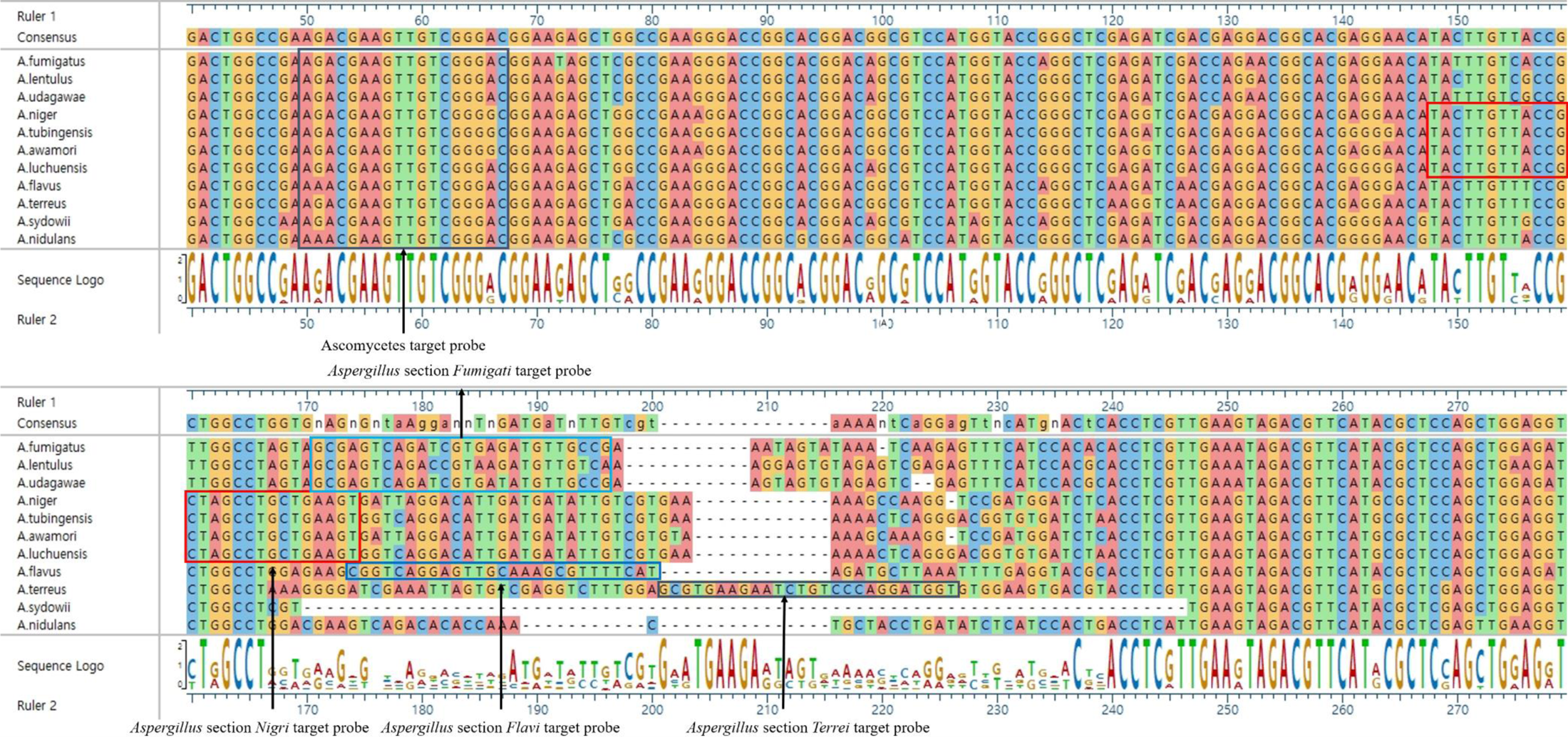

Supplement: S1 Fig — The MegAlign Pro program from DNAStar Lasergene (version 15 software package) was used to compare the sequence analysis by using Clustal Omega alignment. The comparison of benA sequences in Aspergillus species. All benA sequences in Aspergillus were compared and regions specific to the section without affecting other sections were selected as probe candidates, and primers that included all selected regions were chosen. Finally, one pair of primers and five probes were selected. (TIF) [file pone.0229561.s001.tif]

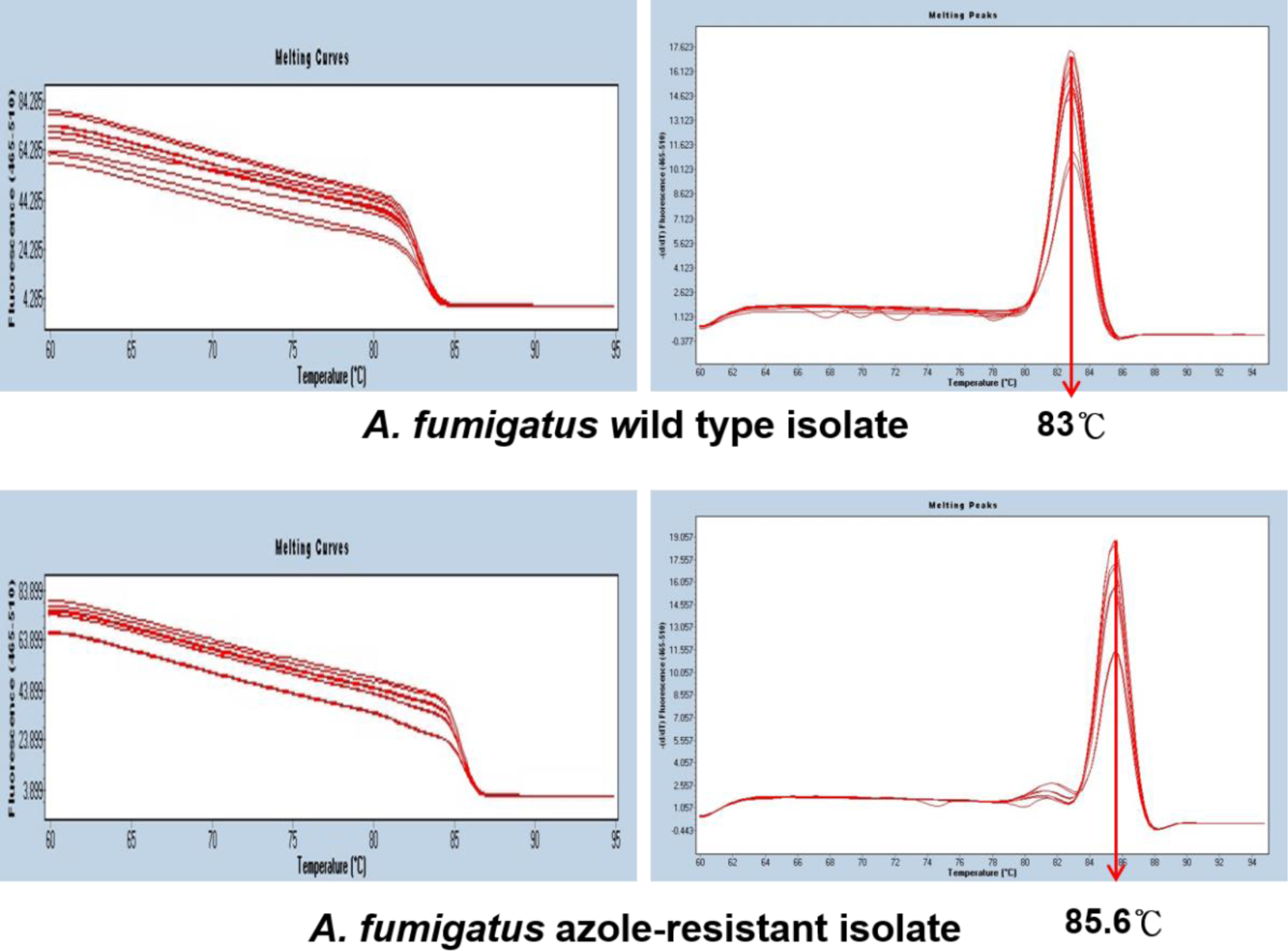

Supplement: S2 Fig — Wild-type (WT) isolate without azole resistance in A. fumigatus and azole-resistant isolate with TR34 sequence were used in the experiment. A pair of primers that amplified the region in the cyp51A promotor known to be involved in azole resistance was used for the melting peak analysis. DNA was subjected to a 10-fold dilution (4 ng to 40 fg). The melting temperatures from the melting curve analysis were different: 83.0ºC ± 0.3ºC in WT and 85.6ºC ± 0.6ºC in azole-resistant type (n = 3). (TIF) [file pone.0229561.s002.tif]

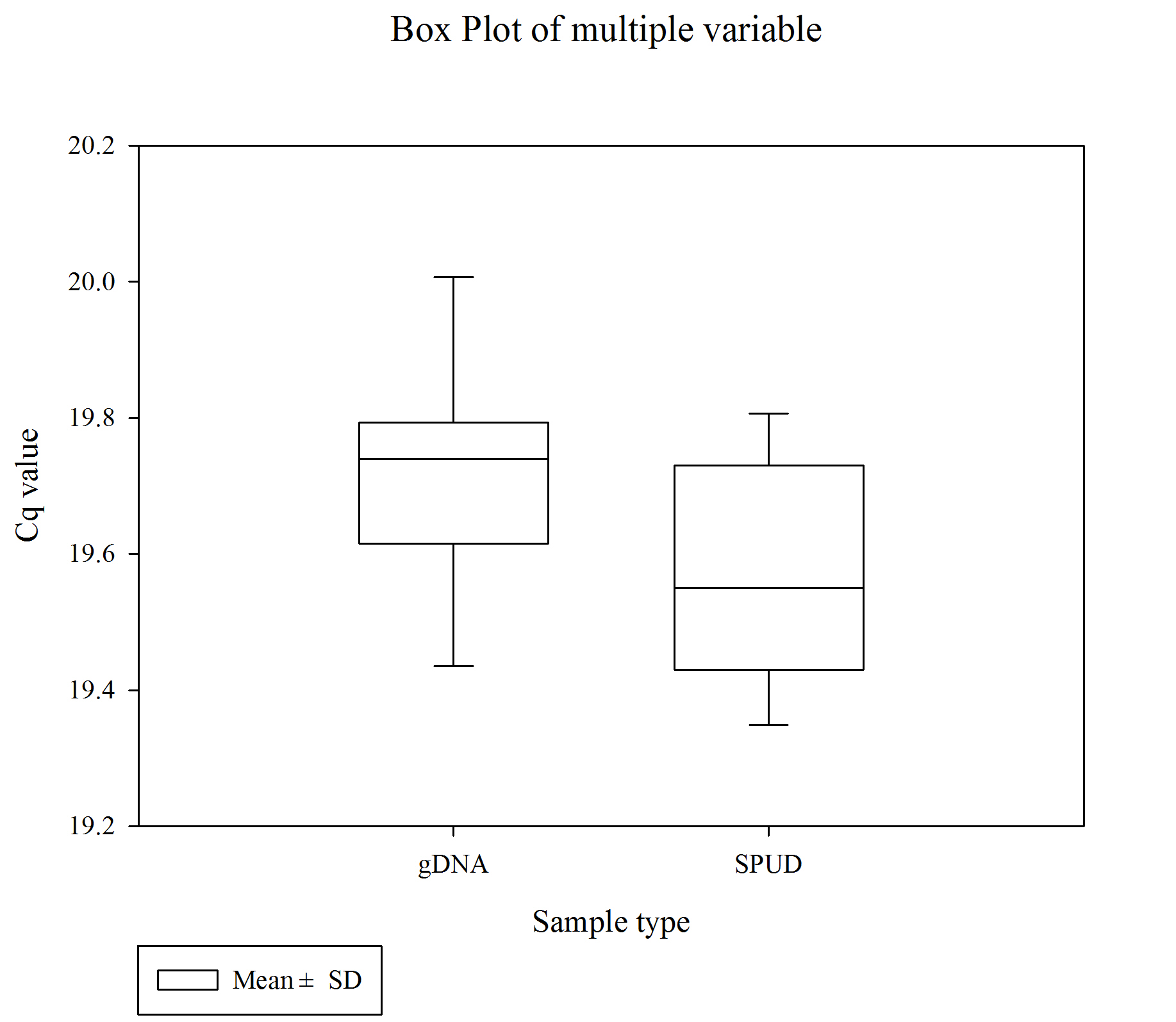

Supplement: S3 Fig — Quantitative PCR was performed using positive control SPUD plasmid DNA (1.3 × 10^5^ copies / μL, n = 20) and various genomic DNAs containing the same amount of SPUD (n = 70), and the results were plotted on a box plot. It can be seen that the Cq value between the two DNAs varies within 1 cycle. The experiment repeated three times. (JPG) [file pone.0229561.s003.JPG]
