# Supplementary material for: Development of multiplex real-time PCR for rapid identification and quantitative analysis of Aspergillus species
Source: PLoS One. 2020 Mar 9;15(3):e0229561. doi: 10.1371/journal.pone.0229561 (PMC7062252; doi:10.1371/journal.pone.0229561)
Supplement: S1 Table — (DOCX) [file pone.0229561.s004.docx]

**S1 Table. Non-*Aspergillus* species used for negative control in qualitative analysis.**

| No. | Species | No. | Species | No. | Species |
| --- | --- | --- | --- | --- | --- |
| 1 | *Alternaria alternata* | 17 | *Paecilomyces formosus* | 33 | *Talaromyces diversus* |
| 2 | *Alternaria arborescens* | 18 | *Paecilomyces variotii* | 34 | *Talaromyces subtropicalis* |
| 3 | *Byssochlamys nivea* | 19 | *Penicillium chermesinum* | 35 | *Thermoascus crustaceus* |
| 4 | *Byssochlamys spectabilis* | 20 | *Penicillium citreonigrum* | 36 | *Thermomyces lanuginosus* |
| 5 | *Cephalotheca foveolata* | 21 | *Penicillium citrinum* | 37 | *Thielavia terricola* |
| 6 | *Cercospora asparagi* | 22 | *Penicillium hispanicum* | 38 | *Trichoderma harzianum* |
| 7 | *Cercospora cf. malloti* | 23 | *Penicillium oxalicum* | 39 | *Candida albicans* |
| 8 | *Cochliobolus lunatus* | 24 | *Penicillium rubens* | 40 | *Schizophyllum commune* |
| 9 | *Curvularia hawaiiensis* | 25 | *Penicillium rubidurum* | 41 | *Corticiaceae sp.* |
| 10 | *Curvularia lycopersici* | 26 | *Periconia sp.* | 42 | *Ganoderma carnosum* |
| 11 | *Fusarium denticulatum* | 27 | *Purpureocillium lilacinum* | 43 | *Gloeophyllum trabeum* |
| 12 | *Fusarium fujikuroi* | 28 | *Scedosporium aurantiacum* | 44 | *Irpex lacteus* |
| 13 | *Fusarium proliferatum* | 29 | *Sordariomycetidae sp.* | 45 | *Phlebia tremellosa* |
| 14 | *Fusarium solani* | 30 | *Talaromyces pinophilus* | 46 | *Porostereum spadiceum* |
| 15 | *Hamigera insecticola* | 31 | *Talaromyces columbinus* | 47 | *Rhizomucor pusillus* |
| 16 | *Leiotrametes lactinea* | 32 | *Talaromyces stollii* |  |  |
