# Supplementary material for: Development of multiplex real-time PCR for rapid identification and quantitative analysis of Aspergillus species
Source: PLoS One. 2020 Mar 9;15(3):e0229561. doi: 10.1371/journal.pone.0229561 (PMC7062252; doi:10.1371/journal.pone.0229561)
Supplement: Attachment — Submitted filename: PONE-D-19-32117_reviewer.pdf [file pone.0229561.s006.pdf]

PLOS ONE

Development of Multiplex Real-time PCR for Rapid Identification and Quantitative Analysis of Aspergillus Species

--Manuscript Draft--

**Manuscript Number:**	PONE-D-19-32117
**Article Type:**	Research Article
**Full Title:**	Development of Multiplex Real-time PCR for Rapid Identification and Quantitative Analysis of Aspergillus Species
**Short Title:**	Multiplex real-time PCR for Aspergillus species
**Corresponding Author:**	Dong-Gun Lee<br>College of Medicine, The Catholic University of Korea<br>Seoul, KOREA, REPUBLIC OF
**Keywords:**	Aspergillus; Drug Resistance, Fungal; Multiplex Polymerase Chain Reaction; Early Diagnosis; DNA Probes
**Abstract:**	The identification of Aspergillus species and azole resistance is highly important for the treatment of invasive aspergillosis (IA), which requires improvements in current fungal diagnostic methods. We aimed to develop multiplex real-time PCR to identify major Aspergillus section and azole resistance. BenA and cyp51A genes were used to design primers, probes, and control DNA for multiplex PCR. Qualitative and quantitative analysis was conducted for 71 Aspergillus and 47 non-Aspergillus isolates. Further, the limit of detection (LOD) and limit of quantitation (LOQ) from hyphae or conidia were determined according to the culture time. Newly developed real-time PCR showed 100% specificity to each Aspergillus section (Fumigati, Nigri, Flavi, and Terrei), without cross-reaction between different sections. In quantitative analysis of sensitivity measurements, LOD and LOQ were 40 fg and 400 fg, respectively. Melting temperature analysis of the cyp51A promoter to identify azole resistance showed temperatures of 83.0 ± 0.3°C and 85.6 ± 0.6°C for susceptible A. fumigatus and resistant isolates with TR34 mutation, respectively. The minimum culture time and fungal colony size required for successful detection were 24 h and 0.4 cm in diameter, respectively. The developed multiplex real-time PCR can identify common Aspergillus sections quantitatively and detect presence of the TR34 mutation. Further, this method shows high sensitivity and specificity, allowing successful detection of early-stage fungal colonies within a day of incubation. These results can provide a template for rapid and accurate diagnosis of IA.
**Order of Authors:**	Won-Bok Kim
	Chulmin Park
	Sung-Yeon Cho
	Hye-Sun Chun
	Dong-Gun Lee
**Additional Information:**	
**Question**	**Response**
**Financial Disclosure**<br><br>Enter a financial disclosure statement that describes the sources of funding for the work included in this submission. Review the submission guidelines for detailed requirements. View published research articles from *PLOS ONE* for specific examples.	This research was supported by an industry-university research grant (5-2019-D0166-00004) through the Yuhan Corporation.

This statement is required for submission and **will appear in the published article** if the submission is accepted. Please make sure it is accurate.<br><br>**Unfunded studies**<br>Enter: *The author(s) received no specific funding for this work.*<br><br>**Funded studies**<br>Enter a statement with the following details:<br>• Initials of the authors who received each award<br>• Grant numbers awarded to each author<br>• The full name of each funder<br>• URL of each funder website<br>• Did the sponsors or funders play any role in the study design, data collection and analysis, decision to publish, or preparation of the manuscript?<br>• **NO** - Include this sentence at the end of your statement: *The funders had no role in study design, data collection and analysis, decision to publish, or preparation of the manuscript.*<br>• **YES** - Specify the role(s) played.<br><br>* typeset	
**Competing Interests**<br><br>Use the instructions below to enter a competing interest statement for this submission. On behalf of all authors, disclose any competing interests that could be perceived to bias this work—acknowledging all financial support and any other relevant financial or non-financial competing interests.<br><br>This statement **will appear in the published article** if the submission is accepted. Please make sure it is accurate. View published research articles from *PLOS ONE* for specific examples.	The authors have declared that no competing interests exist.

**NO authors have competing interests**<br><br>Enter: *The authors have declared that no competing interests exist.*<br><br>**Authors with competing interests**<br><br>Enter competing interest details beginning with this statement:<br><br>*I have read the journal's policy and the authors of this manuscript have the following competing interests: [insert competing interests here]*<br><br>* typeset	
**Ethics Statement**<br><br>Enter an ethics statement for this submission. This statement is required if the study involved:<br><br>• Human participants<br>• Human specimens or tissue<br>• Vertebrate animals or cephalopods<br>• Vertebrate embryos or tissues<br>• Field research<br><br>Write "N/A" if the submission does not require an ethics statement.<br><br>General guidance is provided below. Consult the submission guidelines for detailed instructions. **Make sure that all information entered here is included in the Methods section of the manuscript.**	N / A

Powered by Editorial Manager® and ProduXion Manager® from Aries Systems Corporation

**Format for specific study types**<br><br>**Human Subject Research (involving human participants and/or tissue)**<br>• Give the name of the institutional review board or ethics committee that approved the study<br>• Include the approval number and/or a statement indicating approval of this research<br>• Indicate the form of consent obtained (written/oral) or the reason that consent was not obtained (e.g. the data were analyzed anonymously)<br><br>**Animal Research (involving vertebrate animals, embryos or tissues)**<br>• Provide the name of the Institutional Animal Care and Use Committee (IACUC) or other relevant ethics board that reviewed the study protocol, and indicate whether they approved this research or granted a formal waiver of ethical approval<br>• Include an approval number if one was obtained<br>• If the study involved *non-human primates*, add *additional details* about animal welfare and steps taken to ameliorate suffering<br>• If anesthesia, euthanasia, or any kind of animal sacrifice is part of the study, include briefly which substances and/or methods were applied<br><br>**Field Research**<br><br>Include the following details if this study involves the collection of plant, animal, or other materials from a natural setting:<br>• Field permit number<br>• Name of the institution or relevant body that granted permission	
**Data Availability**<br><br>Authors are required to make all data underlying the findings described fully available, without restriction, and from the time of publication. PLOS allows rare exceptions to address legal and ethical concerns. See the PLOS Data Policy and FAQ for detailed information.	Yes - all data are fully available without restriction

A Data Availability Statement describing where the data can be found is required at submission. Your answers to this question constitute the Data Availability Statement and **will be published in the article**, if accepted.<br><br>**Important:** Stating 'data available on request from the author' is not sufficient. If your data are only available upon request, select 'No' for the first question and explain your exceptional situation in the text box.<br><br>Do the authors confirm that all data underlying the findings described in their manuscript are fully available without restriction?	
**Describe where the data may be found in full sentences. If you are copying our sample text, replace any instances of XXX with the appropriate details.**<br><br>• If the data are **held or will be held in a public repository**, include URLs, accession numbers or DOIs. If this information will only be available after acceptance, indicate this by ticking the box below. For example: *All XXX files are available from the XXX database (accession number(s) XXX, XXX.).*<br>• If the data are all contained **within the manuscript and/or Supporting Information files**, enter the following: *All relevant data are within the manuscript and its Supporting Information files.*<br>• If neither of these applies but you are able to provide **details of access elsewhere**, with or without limitations, please do so. For example:<br><br>*Data cannot be shared publicly because of [XXX]. Data are available from the XXX Institutional Data Access / Ethics Committee (contact via XXX) for researchers who meet the criteria for access to confidential data.*<br><br>*The data underlying the results presented in the study are available from (include the name of the third party*	All relevant data are within the manuscript and its Supporting Information files.

and contact information or URL).<br>• This text is appropriate if the data are owned by a third party and authors do not have permission to share the data.<br><br>* typeset	
Additional data availability information:	

Manuscript
Click here to download Manuscript Development of Multiplex Real-time PCR [Plos One].docx

Development of multiplex real-time PCR for rapid identification and quantitative analysis of *Aspergillus* species

Running title: Multiplex real-time PCR for *Aspergillus* species

Won-Bok Kim[1], Chulmin Park[1], Sung-Yeon Cho[1,2,3], Hye-Sun Chun[1], Dong-Gun Lee[1,2,3*]

[1]Vaccine Bio Research Institute, College of Medicine, The Catholic University of Korea,

[2]Division of Infectious Diseases, Department of Internal Medicine, College of Medicine, The Catholic University of Korea,

[3]Catholic Hematology Hospital, Seoul St. Mary's Hospital, College of Medicine, The Catholic University of Korea, Seoul, Korea

***Corresponding author**

E-mail: symonlee@catholic.ac.kr (DGL)

Abstract

The identification of *Aspergillus* species and azole resistance is highly important for the treatment of invasive aspergillosis (IA), which requires improvements in current fungal diagnostic methods. We aimed to develop multiplex real-time PCR to identify major *Aspergillus* section and azole resistance. *BenA* and *cyp51A* genes were used to design primers, probes, and control DNA for multiplex PCR. Qualitative and quantitative analysis was conducted for 71 *Aspergillus* and 47 non-*Aspergillus* isolates. Further, the limit of detection (LOD) and limit of quantitation (LOQ) from hyphae or conidia were determined according to the culture time. Newly developed real-time PCR showed 100% specificity to each *Aspergillus* section (*Fumigati*, *Nigri*, *Flavi*, and *Terrei*), without cross-reaction between different sections. In quantitative analysis of sensitivity measurements, LOD and LOQ were 40 fg and 400 fg, respectively. Melting temperature analysis of the *cyp51A* promoter to identify azole resistance showed temperatures of 83.0 ± 0.3°C and 85.6 ± 0.6°C for susceptible *A. fumigatus* and resistant isolates with TR34 mutation, respectively. The minimum culture time and fungal colony size required for successful detection were 24 h and 0.4 cm in diameter, respectively. The developed multiplex real-time PCR can identify common *Aspergillus* sections quantitatively and detect presence of the TR34 mutation. Further, this method shows high sensitivity and specificity, allowing successful detection of early-stage fungal colonies within a day of incubation. These results can provide a template for rapid and accurate diagnosis of IA.

Keywords: *Aspergillus*; Drug Resistance, Fungal; Multiplex Polymerase Chain Reaction; Early Diagnosis; DNA Probes

Introduction

Invasive aspergillosis (IA) is a fatal disease caused by *Aspergillus* species that occurs mainly in immunocompromised patients [1]. Common species that cause IA include *Aspergillus fumigatus, A. flavus, A. terreus,* and *A. niger*. Recently, global studies have reported "cryptic species" such as *A. lentulus, A. udagawae,* and *A. tubingensis* and increasing antifungal resistance. Above all, rapid and accurate fungal diagnosis is important, as the morbidity and mortality of IA remain high, and diagnosis and treatment impart a significant economic burden [2-5].

The method of *Aspergillus* species identification includes morphologic identification of fungi through culture and molecular identification via the polymerase chain reaction (PCR) [5, 6]. The former method is currently the gold standard; however, its use can depend largely on clinical specimen quality and the proficiency of the microbiology test personnel [6, 7] The latter method, molecular identification of filamentous Ascomycetes, is mainly conducted through sequence analysis of the internal transcribed spacer (*ITS*) region, and *β-tubulin* (*benA*) and *calmodulin* (*CaM*) genes are also used for cryptic species-level identification [2, 8, 9]. However, such molecular identification is limited, as it takes 7–14 days to reach the final diagnosis; specifically, it takes 3–10 days to produce conidia following fungal culture from the clinical sample, followed by an additional 2–3 days to extract DNA from the conidia and obtain sequencing analysis results [6, 10]

Therefore, to take advantage and overcome the shortcomings of the multi-step molecular diagnosis method, there have been many efforts to explore new diagnostic approaches [6-13]. In this study, we aimed to develop a method for rapid molecular identification by using a multiplex platform that allows for quantitative analysis of major *Aspergillus* sections and rapid detection of azole resistance.

Material & method

Fungal isolates and culture

The fungal isolates used in this study were representative strains of clinical and environmental isolates stored anonymously, and standard strains including *A. fumigatus* (ATCC 16424; American Type Culture Collection, Manassas, VA, USA), *A. terreus* (ATCC 10690), *A. flavus* (ATCC 16883), and *A. niger* (ATCC 16888) [2, 14]. The representative *Aspergillus* isolates were selected for each sequence type according to *benA* and *cyp51A* sequencing results. The *Aspergillus* isolates used in this study included 20 strains of *A. fumigatus,* 2 strains of *A. lentulus,* 1 strain of *A. turcosus,* 1 strain of *A. udagawae,* 2 strains of *A. luchuensis,* 11 strains of *A. awamori,* 11 strains of *A. niger,* 12 strains of *A. tubingensis,* 4 strains of *A. flavus,* 1 strain of *A. nidulans,* 2 strains of *A. sydowii,* 3 strains of *A. terreus,* and 1 strain of *A. subramanianii*, which were registered to GenBank in our previous study [2]. In addition, 38 non-*Aspergillus* filamentous Ascomycetes (No. 1–38), 1 non-filamentous Ascomycetes (No. 39), and 8 non-Ascomycetes molds (No. 40–47) were used to measure the specificity of the developed molecular identification method (Supplementary Table 1). Fungal isolates were cultured using Sabouraud's dextrose agar medium (Becton-Dickinson Labware, Franklin Lakes, NJ, USA) in an incubator at 35°C for 1–10 days. Conidia or hyphae were harvested for genomic DNA (gDNA) extraction using 0.85% NaCl with 0.05% Tween 20. Pellets of conidia or hyphae were stored at −80°C until DNA extraction. The Institutional Review Board of Seoul St. Mary's Hospital approved the research protocol of this study (KC16SISI0307) and waived informed consent for use of fungal isolates stored anonymized.

DNA extraction and purification

To optimize DNA extraction from fungal isolates, the following steps were conducted. In brief, the hyphae pellets stored in the deep freezer were placed in 500 µL Trizol reagent (Invitrogen, Carlsbad, CA, USA), and the conidia pellets were placed in 500 µL of yeast cell lysis solution (MasterPure™ Yeast DNA Purification Kit, Epicentre, Madison, WI, USA); subsequently, the pellets were subjected to two freeze-thaw cycles. After adding RNase A (Epicentre), incubating at 5 min in room temperature. And bead-beating (1.0 mm zirconium beads; Sigma-Aldrich, St. Louis, MO, USA) was repeated 3–5 times at 12,500 rpm for 5 min and cooled on ice at 1 min in between time. Then, the supernatant was obtained after centrifugation (>12,000 × *g*, 4°C, 5 min). In the method using Trizol

reagent, chloroform (Sigma-Aldrich) was added and reacted at room temperature for 5 min; in the method using yeast cell lysis solution, MPC protein precipitation reagent (Epicentre) was added and vortexed. The sample was centrifuged (>12,000 × *g*, 4°C, 15 min) to collect the supernatant, which was then treated with isopropanol (Sigma-Aldrich). A QIAamp DNA Mini spin column (Qiagen, Hildren, Germany) was used to purify fungal DNA. The extracted DNA concentration was measured using Quant-iT™ PicoGreen™ dsDNA Reagent (Invitrogen) according to the manufacturer's protocol. In the case that further DNA purification was required, the DNeasy PowerClean Pro CleanUp Kit (Qiagen) was used according to the manufacturer's protocol. The extracted DNA was stored at −20°C before the next experiment.

Design of primers, probes, and synthetic control DNA

In this study, the sequences of clinical and environmental *Aspergillus* isolates were analyzed to design new primers for amplifying the relatively short sequence of exons 5–6 of the *benA* gene (for *A. fumigatus* AF293 strain *benA* gene, GCA_000002655.1_ASM265v1, locus_tag = AFUA_1G10910, location no. 544-803) (Table 1). The nucleotide sequence of *Aspergillus* isolates was analyzed using MegAlign Pro 15 (DNASTAR, Madison, WI, USA). A probe that could detect filamentous Ascomycetes was designed in exon 5, and other probes were designed for the intron between exons 5 and 6 (Supplementary Figure 1). For the amplification and simultaneous detection of the *benA* target, dimer and hairpin formation of primers and probes was measured, and heterodimer delta G values ranged from −3.6 to −8.9 kcal/mol of five probe.

Table 1. Nucleic acid sequences of biomarkers (primers or/and probes set) designed for multiplex identification in this study.

PCR	Target species	Primers / probes	Sequence (5′ → 3′)
Multiplex real-time PCR primer	*Aspergillus spp.*	benA F3	TCGGTGTAGTGACCCTTGG
		benA R2	GCTGGAGCGYATGAACGTCT
	A. tubingensis	Tubcyp 1F46	CTCGTTGCGATAGTCTTKAATGT
		Tubcyp 1R308	GCCCAAGTATACGGTKGTCTTTT
	A. fumigatus	TR F1	TAATCGCAGCACCACTTCAG
		TR R1	AGGGTGTATGGTATGCTGGAA
Hydrolysis probe	Ascomycetes	Asco 1F9	6-FAM-AVACGAAGTTGTCGGGRC-TAMRA
	Section *Fumigati*	Fumi 1R2	HEX-CGGCAACATCTCACGATCTGACTCGC-BHQ1

	Section *Nigri*	Nig 1R26	6-FAM-ACTTCAGCAGGCTAGCGGTAACAAGT-TAMRA
	Section *Flavi*	Flavi 1F18	HEX-CGGTCAGGAGTTGCAAAGCGTTTTCA-BHQ1
	Section *Terrei*	Terrei 1R29	6-FAM-ACCATCCTGGGACAGATTCTYCACGC-TAMRA
	A. tubingensis	Tubcyp 201	6-FAM-GTCAGYCACTGTCCATATGCGATTG-TAMRA[†]

[†] The underlined area is a probe synthesized by LNA.

[‡] Wild type

We designed two staged probe sets: For the first stage, primer and probe sequences specific to four major *Aspergillus* sections (*Fumigati*, *Nigri*, *Terrei*, and *Flavi*) were designed, and a control probe sequence was also determined to identify filamentous Ascomycetes. Based on the analysis using the OligoAnalyzer web program (https://sg.idtdna.com/pages/tools/oligoanalyzer) for each primer and probe, the final biomarkers were developed for multiplex molecular identification (Table 1). The specificity of the synthesized probe was evaluated using BLASTn (https://blast.ncbi.nlm.nih.gov/Blast.cgi), and the presence of cross-reactions between sections was identified. For the second stage, primers and probes specific for both TR mutations in the *cyp51A* promoter and *A. tubingensis* were designed (Table 1). The *A. tubingensis* probe was designed for a specific sequence in the *cyp51A* gene using locked nucleic acid to increase specificity. Promoter mutation was detected by melting temperature analysis instead of the probe detection method.

Further, synthetic control DNA was designed as a control for *benA* multiplex real-time PCR to measure specificity and sensitivity and used for absolute quantitation by a standard curve. Control DNA that included specific probes and primer sequences for each *Aspergillus* section was synthesized (Table 2). The synthesized control DNA was cloned into pUC57 plasmid (Bionics co., Seoul, Korea) and then transformed into *Escherichia coli* by heat-shock method. The transformed *E. coli* were stored as stock, and the plasmid DNA was amplified and used in the experiment.

Table 2. Positive control nucleic acid sequences for *Aspergillus* real-time PCR assays

Oligo	DNA Sequence (5′ → 3′)
Aspergillus Positive Control DNA (276 bp)	ATCGTAAGCTTTCGGTGTAGTGACCCTTGGCCCAGCCGAAGACGAAGTTGTCGGGACC<br>benA F3 primer Asco 1F9 probe<br>TTTTCAGCACGGACAACTCAGGGACGGTGTGATCTAACCTCATGGTACGCGTGGAGAA<br>Terrei<br>CACGAGGTAGCGAGTCAGATCGTGAGATGTTGCCGAAATAGTATAAATCAAGAGACTT<br>Fumi 1R2 probe<br>ATAGACGTTCATGCGCTCCAGCGAGCTCATCGT<br>benA R2 primer

Qualitative and quantitative analysis by multiplex real-time PCR

Qualitative and quantitative analyses were conducted using the LightCycler® 480 Probes Master Kit (Roche, Basel, Switzerland) and Roche LightCycler® 480 Instrument II (Roche). The primer and probe concentrations used in the experiment were 0.5 μM and 0.1 μM, respectively. The amplification process was conducted as follows: 10 min of pre-denaturation at 95°C followed by 40 cycles of denaturation at 95°C for 25 s, annealing at 58°C for 30 s, and extension at 72°C for 35 s. For quantitative analysis, 4 ng fungal gDNA and 4 pg *Aspergillus* positive control DNA were each subjected to 10-fold serial dilutions to reach 4 fg/μL and 4 ag/μL, respectively. Melting peak analysis was conducted using the LightCycler® 480 SYBR Green I Master Kit (Roche) and Roche LightCycler® 480 Instrument II (Roche).

The results were analyzed using LightCycler® 480 Software version 1.5.0 SP3 (Roche). The quantification cycle (Cq) was calculated automatically by the program based on curve fitting to the baseline curve subtraction. A standard curve was obtained using the measured results, and the slope value, mean Cq value (MCq), and standard deviation (SD) were calculated. The amplification efficiency (Efficiency, E [%]) was calculated by the following equation: $[10^{(-1/(\text{slope}))} - 1] \times 100$. Sensitivity analysis of the developed identification method was conducted using the limit of detection (LOD) and limit of quantitation (LOQ). The LOD was set to the minimum value detected above 95% of analysis results (<5% false negative result). The LOQ was determined to be a value of MCq − (2 × SD) below 35 [16, 17].

All experiments were conducted in accordance with the “Minimum Information for the Publication of Real-Time Quantitative PCR Experiments” (MIQE) guidelines [18, 19]. In addition, the experiment was conducted in a biosafety cabinet in an area separate from the DNA extraction area to prevent contamination from aerosol and carryover. To remove contaminants on the surface, 1.5% sodium hypochlorite (NaOCl) was used, followed by 70% EtOH. The experiment was conducted using filter tips (TipOne; StarLab, Hamburg, Germany) after removing all possible contaminants.

Results

Qualitative analysis to determine probe specificity

Multiplex real-time PCR of 118 fungal DNA samples showed that the probes specifically amplified the *Aspergillus* section as presented in Table 3. No amplification was observed in non-*Aspergillus* isolates. Further, the *Aspergillus* positive control DNA was amplified in all primers and probes designed for the first stage, and there was no interference between probes. For the second stage, real-time PCR was performed for 20 *A. fumigatus* (17 wild-type and 3 mutant strains), 2 *A. lentulus*, 1 *A. turcosus*, and 1 *A. udagawae* strains, and the melting peaks were analyzed. The melting temperature was 83.0 ± 0.3°C in *A. fumigatus* wild-type samples and 85.6 ± 0.6°C in mutant isolates with TR34 (Supplementary Figure 2). *Aspergillus* section *Fumigati* isolates that were not *A. fumigatus* were not amplified. In addition, one primer pair (Tubcyp 1F46 and Tubcyp 1R308) and one probe (Tubcyp 201) designed to identify *A. tubingensis* from *Aspergillus* section *Nigri* were tested on 36 *Aspergillus* isolates of section *Nigri* in real-time PCR analysis. All 12 *A. tubingensis* isolates with different *cyp51A* sequence types were amplified (Table 3); other isolates were not amplified.

TABLE 3. Specific amplification of the designed probes

Probe (n)	*Aspergillus* section					Non-*Aspergillus* (47)
	Fumigati (24)	*Nigri* (36)	*Flavi* (4)	*Terrei* (3)	other (4)	
Asco 1F9	24 / 24 (100%)	36 / 36 (100%)	4 / 4 (100%)	3 / 3 (100%)	4 / 4 (100%)	30 / 38[†] (63.82%)
Fumi 1R2	24 / 24 (100%)	0 / 36 (0%)	0 / 4 (0%)	0 / 3 (0%)	0 / 4 (0%)	0 / 47 (0%)
Nig 1R26	0 / 24 (0%)	36 / 36 (100%)	0 / 4 (0%)	0 / 3 (0%)	0 / 4 (0%)	0 / 47 (0%)
Flavi 1F18	0 / 24 (0%)	0 / 36 (0%)	4 / 4 (100%)	0 / 3 (0%)	0 / 4 (0%)	0 / 47 (0%)
Ter 1R29	0 / 24 (0%)	0 / 36 (0%)	0 / 4 (0%)	3 / 3 (100%)	0 / 4 (0%)	0 / 47 (0%)
Tubcyp 201	0 / 24 (0%)	12 / 12 (100%)	0 / 4 (0%)	0 / 3 (0%)	0 / 4 (0%)	0 / 47 (0%)

[†] Ratio of non-*Aspergillus* Filamentous Ascomycetes

Probe sensitivity and efficiency measurement

Quantitative analysis results of gDNA of major *Aspergillus* sections are shown in Fig 1. There was no interference during the amplification of two probes analyzed in the multiplex assay, and gDNA was amplified from 4 ng/μL to 40 fg/μL in all probes. Similarly, amplification occurred without interference in the positive control DNA,

which was amplified from 4 pg/μL to 40 ag/μL in all probes. The standard curve obtained from plotting gDNA concentration on the X-axis and Cq value on the Y-axis yielded slope values, intercept values, and R^2 –in the ranges of −3.2119 to −3.7230, 40.316 to 43.702, and 0.9979 to 0.9998, respectively. The LOD and LOQ of gDNA in all probes was 40 fg/μL (1.0–1.2 × 10^0 genome copies/μL) and 400 fg/μL (1.0–1.2 × 10^1 genome copies/μL), respectively. For positive control DNA, all probes showed similar correlations and regression analysis results, as shown in Table 4. According to the sensitivity analysis results using this data, the LOD and LOQ were calculated as 40 ag/μL (1.2 × 10^1 genome copies/μL) and 400 ag/μL (1.2 × 10^2 genome copies/μL), respectively (Table 4). The quantitative analysis results for *Aspergillus* gDNA and positive control DNA showed similar correlations and regression coefficients for all probes.

Fig 1. Sensitivity measurements of *Aspergillus* gDNA and quantification curve

Aspergillus gDNA and *Aspergillus* positive-control DNA were subjected to multiplex real-time PCR using Fumi 1R2, Nigri 1R26, Flavi 1F18, Terrei 1R29, Asco 1F9 and the results were presented as a standard curve. The spot is the average of Cq ± SD and n = 6.

Abbreviations. gDNA; genomic DNA, SD; Standard Deviation

TABLE 4. Correlation and regression analysis between two DNA.

gDNA / control DNA	Fumi 1R2	Nig 1R26	Flavi 1F18	Ter 1R29	Asco 1F9
4 ng / 4 pg	21.07 / 21.50	21.01 / 20.12	20.94 / 21.92	20.98 / 21.20	20.93 / 22.08
400 pg / 400 fg	24.30 / 25.21	25.21 / 24.41	24.59 / 25.00	24.31 / 24.41	24.50 / 25.45
40 pg / 40 fg	28.34 / 29.01	29.17 / 28.70	28.07 / 28.42	27.41 / 27.26	27.88 / 28.15
4 pg / 4 fg	31.65 / 32.55	32.53 / 32.00	31.41 / 32.41	30.79 / 30.54	30.83 / 31.37
400 fg / 400 ag	35.05 / 36.23	36.47 / 36.06	35.29 / 35.58	33.94 / 33.58	33.59 / 34.57
40 fg / 40 ag	38.64 / 39.69	39.65 / 39.12	38.44 / 39.10	37.01 / 36.95	37.64 / 36.52

Sensitivity measurement for molecular identification method by culture time

Four reference strains were used to measure the fungal colony diameter over the culture time. The size of *A. fumigatus, A. niger, A. terreus,* and *A. flavus* colonies was 0.2–1.5 cm at 18–24 h after incubation, 1.4–5.2 cm after 48 h, and 4.0–7.8 cm after 72 h. Further, conidia formation was observed after 72 h. Multiplex real-time PCR

using DNA extracted from early-stage colonies showed successful amplification in hyphae-state after culturing for 24 h. The minimum colony diameter to confirm colony DNA amplification in all probes was 0.4 cm (confidence interval, CI > 95%), and the minimum culture time was 18–24 h. The result of multiplex real-time PCR of DNA from hyphae-state colonies of 0.4 cm in diameter for all isolates showed Cq values of 31.425 ± 0.245 for *A. fumigatus*, 32.345 ± 0.185 for *A. niger*, 32.63 ± 0.36 for *A. terreus*, and 32.16 ± 0.12 for *A. flavus*. This suggested that amplification occurred within the range of the standard curve of *Aspergillus* positive control DNA used as standard DNA, which is between approximately 1.0–1.2 × 10^2 and 1.0–1.2 × 10^1 copies/μL when calculated with respect to genomic DNA copy number.

Discussion

In this study, a molecular identification method for major *Aspergillus* sections (*Fumigati, Nigri, Flavi,* and *Terrei*) was developed based on the *benA* gene, which reduced diagnosis time compared with the current methods of morphological or molecular identification based on ITS sequencing. In addition, TR mutation and the *cyp51A* gene were used to identify azole-resistant *A. fumigatus* and *A. tubingensis*. Instead of probe-based sequencing, melting temperature analysis methods were selected for faster and simultaneous analysis. The designed probes and primers were used for qualitative and quantitative analyses of 118 clinical and environmental isolates. Sensitivity and specificity were evaluated for the developed multiplex real-time PCR method. Further, both LOD and LOQ with minimum culture time were analyzed to evaluate to the applicability of the method for early diagnosis.

For the molecular identification of mold, there is a need to improve the process and purity of DNA extraction. Extracted DNA quality and impurity contents, such as PCR inhibitors, differ between each clinical and environmental isolate; thus, it is difficult to conduct quantitative measurements and evaluate the sensitivity of molecular identification. In this study, we optimized methods to extract mold DNA. First, freeze-thawing and bead-beating methods were used for mechanical lysis, and different lysis and protein removal solutions were used for the conidia and hyphae (hyphae, Trizol solution and isopropanol; conidia, Epicentre's lysis buffer and protein precipitation solution). Different lysis buffers were used because Trizol reagent (Invitrogen) led to a better yield in the hyphae but affected the purity in conidia, as the handling process during supernatant isolation was challenging due to difficulty in distinguishing the supernatant. In the purification process, the most commonly used method was column chromatography for final purification, and high-purity fungal DNA was obtained in almost all strains. For accurate quantification, Quant-iT™ PicoGreen™ dsDNA Reagent (Invitrogen) was used, which has a lower error rate compared with conventional OD methods and offers advantages in quantitative analysis and sensitivity measurements.

To establish a multiplex real-time PCR method with high specificity and sensitivity, *benA* and *cyp51A* were used to select a primer pair and probe specific to the major *Aspergillus* sections, and *benA* has been used as an important molecular marker to analyze the phylogenic relationship between filamentous Ascomycetes [5, 20]. The genetic analysis of *benA* is useful to distinguish cryptic species, which is not possible with ITS sequencing. Although there are differences between species, approximately 560 bp (bt2ab region: exons 3−6) can be amplified for nucleotide sequence analysis. However, in this study, the region was designed to be shorter (exons 5−6) to be

more suitable for the multiplex probe-based real-time PCR method, which improved efficiency while maintaining specificity (Supplementary Figure 1, Table 1). While developing a probe-based method for rapid simultaneous detection, we also designed *benA* primers for further sequence analysis to achieve specificity toward each species after amplification/probe detection. This could specifically amplify certain *Aspergillus* sections through multiplex real-time PCR, reflecting high specificity without cross-amplification of other fungal species. Further, quantification analysis of each probe showed an extremely small error rate, and approximately 10^1 genomic copies could be detected, indicating high sensitivity. These results show that the developed method have similar sensitivity to existing methods used for detection and quantification analysis [4, 18].

Regarding the use of standard DNA as a control for a developed method, gDNA usage for each *Aspergillus* strain is complex and requires much time; furthermore, many standard DNA types for specific probes are needed. Therefore, in this study, synthetic DNA was designed and used as standard DNA that reacted with all probes. The developed *Aspergillus* positive control DNA was amplified in all *benA* probes that identified different *Aspergillus* sections. Further, reconstructed *Aspergillus* positive control DNA for quantitative analysis was used to compare analyses with a standard curve, which correlated with the standard gDNA curve (Table 4). These results indicate that this DNA can be used as the control for sample gDNA quantification, to verify the primers and probe used in the experiment, and to measure experimental sensitivity and specificity. The synthetic DNA could be applied with the newly designed *benA* probe in the field for IA diagnosis.

Among the sections amplified in the first stage, azole resistance in *A. fumigatus* and *A. tubingensis* of section *Nigri* can be detected using multiplex real-time PCR primer/probes designed in the second stage. Using isolates from *Aspergillus* section *Fumigati,* the melting peak and temperature were identified for the primers that specifically amplified the *cyp51A* TR region in *A. fumigatus* and gDNA subjected to a 10-fold serial dilution. Wild-type isolates and mutant isolates with TR34 showed different melting temperatures. In particular, *A. tubingensis* revealed intrinsically higher azole minimum inhibitory concentrations according to previous studies [2, 3, 5]. The *A. tubingensis*-specific primers and probe designed in this study showed that only *A. tubingensis* was amplified without affecting other *Aspergillus* in section *Nigri.*

Interestingly, we tried to use DNA extracted from early-stage fungal colonies for PCR analysis and determined that the minimum diameter and culture time required for successful PCR detection were 0.4 cm and 24 h, respectively. The same results were obtained for four probes, suggesting that fungal colony hyphae cultured for 24 h can be used for earlier molecular identification. Although there may be some limitations in early diagnosis,

our developed method can dramatically reduce the minimum culture period for molecular identification within 2–3 days.

In conclusion, first, newly designed primers and probes were used to conduct a multiplex real-time PCR assay, and high sensitivity and specificity was confirmed through clinical and environmental fungal isolates. Second, azole-resistant *A. fumigatus* and *A. tubingensis* were detected, and major *Aspergillus* sections were identified. Third, this real-time PCR method resulted in successful amplification specific to each section with DNA extracted from *Aspergillus* hyphae in the early phases of the cultured colony. This method can provide a template for rapid and quantitative diagnosis of IA. Fourth, *Aspergillus* positive control DNA designed for the standard control permitted quantitative analysis. It is expected that further research on new biomarkers will be conducted using the database obtained through this study.

Conflict of interest

DGL has served as a consultant for Astellas, MSD, Pfizer, Gilead, and Yuhan; has served as a board member for Astellas, MSD, Gilead and Yuhan; and has received research support, travel support and payment for lectures, including service on Speaker's bureaus, from Astellas, MSD, Pfizer, Gilead, and Yuhan, outside the submitted work. SYC. has received research support and payment for lectures from MSD, Gilead, and Astellas, outside the submitted work. DGL, WBK, CP and SYC have a pending patent application on the development of multiplex real time PCR in Korea.

Author contributions

DGL conceived the idea and led the writing; WBK collected the data and analyzed it and led the writing; CP and SYC analyzed the data and assisted the writing and review processes; HSC collected the data.

References

1. Ullmann AJ, Aguado JM, Arikan Akdagli S, Denning DW, Groll AH, Lagrou K, et al. Diagnosis and management of *Aspergillus* diseases: executive summary of the 2017 ESCMID ECMM ERS guideline. Clinical Microbiology and Infection. 2018;24:e1-e38. doi:https://doi.org/10.1016/j.cmi.2018.01.002
2. Cho SY, Lee DG, Kim WB, Chun HS, Park C, Myong JP, et al. Epidemiology and antifungal susceptibility profile of *Aspergillus* species: comparison between environmental and clinical isolates from patients with hematologic malignancies. J Clin Microbiol. 2019;57(7):e02023-18. doi:10.1128/JCM.02023-18
3. Howard SJ. Multi resistant aspergillosis due to cryptic species. Mycopathologia. 2014;178(5):435-9. doi:10.1007/s11046-014-9774-0
4. Steinbach WJ, Marr KA, Anaissie EJ, Azie N, Quan SP, Meier Kriesche HU, et al. Clinical epidemiology of 960 patients with invasive aspergillosis from the path alliance registry. Journal of Infection. 2012;65(5):453-64. doi:https://doi.org/10.1016/j.jinf.2012.08.003
5. Buil JB, Zoll J, Verweij PE, Melchers WJG. Molecular detection of azole resistant *Aspergillus fumigatus* in clinical samples. Front Microbiol. 2018;9:515-. doi:10.3389/fmicb.2018.00515
6. Buitrago MJ, Aguado JM, Ballen A, Bernal-Martinez L, Prieto M, Garcia-Reyne A, et al. Efficacy of DNA amplification in tissue biopsy samples to improve the detection of invasive fungal disease. Clinical Microbiology and Infection. 2013;19(6):E271-E7. doi:https://doi.org/10.1111/1469-0691.12110
7. Salah H, Lackner M, Houbraken J, Theelen B, Lass-Flörl C, Boekhout T, et al. The emergence of rare clinical *Aspergillus* species in qatar: molecular characterization and antifungal susceptibility profiles. Front Microbiol. 2019;10:1677-. doi:10.3389/fmicb.2019.01677
8. Van Der Linden JWM, Warris A, Verweij PE. *Aspergillus* species intrinsically resistant to antifungal agents. Medical Mycology. 2011;49(Supplement_1):S82-S9. doi:10.3109/13693786.2010.499916
9. Samson RA, Visagie CM, Houbraken J, Hong SB, Hubka V, Klaassen CHW, et al. Phylogeny, identification and nomenclature of the genus *Aspergillus*. Stud Mycol. 2014;78:141-73. doi:10.1016/j.simyco.2014.07.004
10. Hope WW, Walsh TJ, Denning DW. Laboratory diagnosis of invasive aspergillosis. The Lancet Infectious Diseases. 2005;5(10):609-22. doi:https://doi.org/10.1016/S1473-3099(05)70238-3

11. Verweij PE, Ananda-Rajah M, Andes D, Arendrup MC, Brüggemann RJ, Chowdhary A, et al. International expert opinion on the management of infection caused by azole-resistant *Aspergillus fumigatus*. Drug Resistance Updates. 2015;21-22:30-40. doi:https://doi.org/10.1016/j.drup.2015.08.001

12. Verweij PE, Chowdhary A, Melchers WJG, Meis JF. Azole resistance in *Aspergillus fumigatus*: can we retain the clinical use of mold-active antifungal azoles? Clin Infect Dis. 2016;62(3):362-8. doi:10.1093/cid/civ885

13. Hagiwara D, Watanabe A, Kamei K, Goldman GH. Epidemiological and genomic landscape of azole resistance mechanisms in *Aspergillus* fungi. Front Microbiol. 2016;7:1382-. doi:10.3389/fmicb.2016.01382

14. Cho SY, Myong JP, Kim WB, Park C, Lee SJ, Lee SH, et al. Profiles of environmental mold: indoor and outdoor air sampling in a hematology hospital in Seoul, South Korea. Int J Environ Res Public Health. 2018;15(11):2560. doi:10.3390/ijerph15112560

15. Morio F, Aubin GG, Danner-Boucher I, Haloun A, Sacchetto E, Garcia-Hermoso D, et al. High prevalence of triazole resistance in *Aspergillus fumigatus*, especially mediated by TR/L98H, in a French cohort of patients with cystic fibrosis. Journal of Antimicrobial Chemotherapy. 2012;67(8):1870-3. doi:10.1093/jac/dks160

16. Waiblinger H-U, Graf N, Broll H, Grohmann L, Pietsch K. Evaluation of real-time PCR results at the limit of detection. Journal für Verbraucherschutz und Lebensmittelsicherheit. 2011;6(4):411-7. doi:10.1007/s00003-011-0669-4

17. Ferreira T, Farah A, Oliveira TC, Lima IS, Vitório F, Oliveira EMM. Using Real-Time PCR as a tool for monitoring the authenticity of commercial coffees. Food Chemistry. 2016;199:433-8. doi:https://doi.org/10.1016/j.foodchem.2015.12.045

18. Johnson GL, Bibby DF, Wong S, Agrawal SG, Bustin SA. A MIQE-compliant real-time PCR assay for *Aspergillus* detection. PLoS One. 2012;7(7):e40022-e. doi:10.1371/journal.pone.0040022

19. Bustin SA, Benes V, Garson JA, Hellemans J, Huggett J, Kubista M, et al. The MIQE guidelines: minimum information for publication of quantitative real-time PCR experiments. Clinical Chemistry. 2009;55(4):611. doi:10.1373/clinchem.2008.112797

20. Glass NL, Donaldson GC. Development of primer sets designed for use with the PCR to amplify conserved genes from filamentous ascomycetes. Appl Environ Microbiol. 1995;61(4):1323-30.

Supporting information

S1 Fig. Probe and primer design by sequence comparison analysis of major *Aspergillus* species.

The MegAlign Pro program from DNAStar Lasergene (version 15 software package) was used to compare the sequence analysis by using Clustal Omega alignment. The comparison of *benA* sequences in *Aspergillus* species. All *benA* sequences in *Aspergillus* were compared and regions specific to the section without affecting other sections were selected as probe candidates, and primers that included all selected regions were chosen. Finally, one pair of primers and five probes were selected.

S2 Fig. Analysis of melting peak for two types of *Aspergillus fumigatus* showing different azole resistance.

Wild-type (WT) isolate without azole resistance in *A. fumigatus* and azole-resistant isolate with TR34 sequence were used in the experiment. A pair of primers that amplified the region in the *cyp51A* promotor known to be involved in azole resistance was used for the melting peak analysis. DNA was subjected to a 10-fold dilution (4 ng to 40 fg). The melting temperatures from the melting curve analysis were different: 83.0ºC ± 0.3ºC in WT and 85.6ºC ± 0.6ºC in azole-resistant type (n = 3).

Figure 1

Click here to download Figure Fig 1.tif

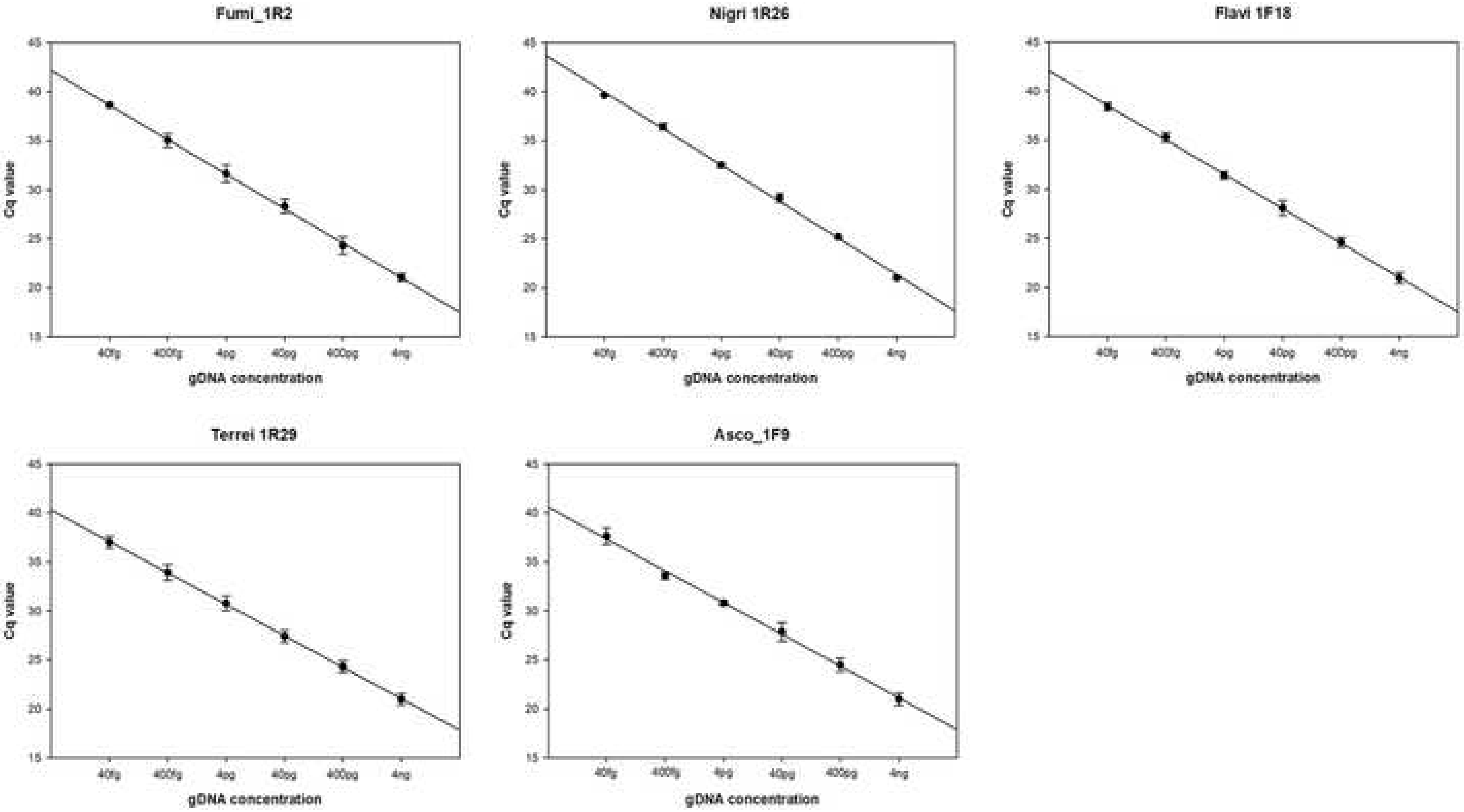

S1 fig

Click here to access/download
Supporting Information
S1 Figure.tif

S2 fig

Click here to access/download

Supporting Information

S2 Figure.tif

S1 Table

Click here to access/download
Supporting Information
S1 Table.docx
