# Supplementary material for: Development of multiplex real-time PCR for rapid identification and quantitative analysis of Aspergillus species
Source: PLoS One. 2020 Mar 9;15(3):e0229561. doi: 10.1371/journal.pone.0229561 (PMC7062252; doi:10.1371/journal.pone.0229561)
Supplement: Attachment — Submitted filename: Dear Editor.docx [file pone.0229561.s005.docx]

Dear Editor;

Thank you for considering me as a reviewer for this publication in your esteemed journal PLOS ONE. The manuscript is well-written and provides information on the use of Taqman real-time PCR in the rapid identification of *Aspergillus* species. However, there are few comments as below:

1. What are the novelties of your work compared to other studies using "multiplex real time PCR" for *Aspergillus* detection like: "Multiplex real-time PCR for detection and quantification of mycotoxigenic Aspergillus, Penicillium and Fusarium" 2009. Journal of Stored Products Research.
2. Lines 56-58, Introduction part: -These steps are shared between your method and "identification via the polymerase chain reaction" methods. - What are the merits of your method compared to other molecular methods such as multiplex PCR?
3. Line 215, discussion part: In average, how much time was reduced compared to other methods?

Sincerely;

Lida Fekrat
