# Supplementary material for: Development of multiplex real-time PCR for rapid identification and quantitative analysis of Aspergillus species
Source: PLoS One. 2020 Mar 9;15(3):e0229561. doi: 10.1371/journal.pone.0229561 (PMC7062252; doi:10.1371/journal.pone.0229561)
Supplement: Attachment — Submitted filename: Plosone revised 18-12-2019.docx [file pone.0229561.s007.docx]

The manuscript is relevant for PlosOne but there are some issues. You have a very good results but I would like that you explain more about material and methods used to synthesize the control DNA. When you explain about the standard curve, never show the efficiency (E). However in M&M is explianed that it has been calcualted.

M&M:

Line 127: What is the protocol used to synthesized the control DNA?

Line 130: why has the author synthesized a control DNA instead to clon different fragments of DNA as positives controls?

Results:

Line 167: delete a "were not"

Line 188: The author should include the Efficiency (%) calculated, as explianed in M&M, to each standard curve.
