# Supplementary material for: Development of multiplex real-time PCR for rapid identification and quantitative analysis of Aspergillus species
Source: PLoS One. 2020 Mar 9;15(3):e0229561. doi: 10.1371/journal.pone.0229561 (PMC7062252; doi:10.1371/journal.pone.0229561)
Supplement: Attachment — Submitted filename: Plosone Response to Reviewers.docx [file pone.0229561.s008.docx]

Dear Editor:

I wish to submit a revised version of the Research Article for publication in *PLOS ONE*, entitled “**Development of Multiplex Real-time PCR for Rapid Identification and Quantitative Analysis of *Aspergillus* Species**” (PONE-D-19-32117).

I thank the editor and reviewers of the ‘*PLOS ONE*’ for taking the time to review our manuscript. I have made some corrections and clarifications in the manuscript after considering the reviewers’ comments.

Below we submit the revised “Competing Interest” our answers to the comments of reviewers. The reviewers’ comments are provided in black color, whereas our answers are given in the **bold letter**. The changes made in the revised manuscript are given in highlight.

**Competing Interest**

This research was supported by an industry-university research grant (5-2019-D0166-00004) through the Yuhan Corporation. The funder provided support in the form of salaries for authors [WBK, CP and HSC], but did not have any additional role in the study design, data collection, and analysis, decision to publish, or preparation of the manuscript. The specific roles of these authors are articulated in the ‘Author contributions’ section. This does not alter our adherence to PLOS ONE policies on sharing data and materials. The authors declare that they have no further competing interests.

We hope that the revised manuscript is suitable for publication in PLOS ONE.

Sincerely,

Dong-Gun Lee, M.D., Ph.D.

Division of Infectious Diseases, Department of Internal Medicine, Vaccine Bio Research Institute, Catholic Hematology Hospital, College of Medicine, The Catholic University of Korea, Seoul, Korea

222, Banpo-daero, Seocho-gu, Seoul St. Mary's Hospital, Seoul, 06591, Korea

E-mail: [symonlee@catholic.ac.kr](mailto:symonlee@catholic.ac.kr), Tel: +82-2-2258-6003

**Answers to Editor’s & Reviewers’ comments**

1. Please ensure that your manuscript meets PLOS ONE's style requirements, including those for file naming. The PLOS ONE style templates can be found at http://www.plosone.org/attachments/PLOSOne_formatting_sample_main_body.pdf and <http://www.plosone.org/attachments/PLOSOne_formatting_sample_title_authors_affiliations.pdf>

**- We made further edits in response to the editor's comments. The revised version was corrected by the style of the journal, and modified parts were given in highlight.**

2. Thank you for stating the following financial disclosure:

"This research was supported by an industry-university research grant (5-2019-D0166-00004) through the Yuhan Corporation."

We note that one or more of the authors have an affiliation to the commercial funders of this research study : [Yuhan Corporation.].

 1) Please provide an amended Funding Statement declaring this commercial affiliation, as well as a statement regarding the Role of Funders in your study. If the funding organization did not play a role in the study design, data collection and analysis, decision to publish, or preparation of the manuscript and only provided financial support in the form of authors' salaries and/or research materials, please review your statements relating to the author contributions, and ensure you have specifically and accurately indicated the role(s) that these authors had in your study. You can update author roles in the Author Contributions section of the online submission form.

* Please also include the following statement within your amended Funding Statement.

“The funder provided support in the form of salaries for authors [insert relevant initials], but did not have any additional role in the study design, data collection and analysis, decision to publish, or preparation of the manuscript. The specific roles of these authors are articulated in the ‘author contributions’ section.”

If your commercial affiliation did play a role in your study, please state and explain this role within your updated Funding Statement.

2) Please also provide an updated Competing Interests Statement declaring this commercial affiliation along with any other relevant declarations relating to employment, consultancy, patents, products in development, or marketed products, etc.

 Within your Competing Interests Statement, please confirm that this commercial affiliation does not alter your adherence to all PLOS ONE policies on sharing data and materials by including the following statement: "This does not alter our adherence to  PLOS ONE policies on sharing data and materials.” (as detailed online in our guide for authors <http://journals.plos.org/plosone/s/competing-interests>). If this adherence statement is not accurate and  there are restrictions on sharing of data and/or materials, please state these. Please note that we cannot proceed with consideration of your article until this information has been declared.

 * Please include both an updated Funding Statement and Competing Interests Statement in your cover letter. We will change the online submission form on your behalf.

Please know it is PLOS ONE policy for corresponding authors to declare, on behalf of all authors, all potential competing interests for the purposes of transparency. PLOS defines a competing interest as anything that interferes with, or could reasonably be perceived as interfering with, the full and objective presentation, peer review, editorial decision-making, or publication of research or non-research articles submitted to one of the journals. Competing interests can be financial or non-financial, professional, or personal. Competing interests can arise in relationship to an organization or another person. Please follow this link to our website for more details on competing interests: <http://journals.plos.org/plosone/s/competing-interests>

**- As you indicated, we revised “Competing Interest” and attached it to the rebuttal letter.**

Review Comments to the Author

Reviewer #1:

1. What are the novelties of your work compared to other studies using "multiplex real time PCR" for *Aspergillus* detection like: "Multiplex real-time PCR for detection and quantification of mycotoxigenic *Aspergillus*, *Penicillium* and *Fusarium*" 2009. Journal of Stored Products Research.

**: I appreciate that you reviewed our paper in detail. In this study, our strategy for the molecular detection of *Aspergillus* focused on the diagnostics of the invasive aspergillosis (IA). Due to the high morbidity and mortality of IA, the rapid detection and identification are very important in the clinical setting. *Aspergillus fumigatus, A. niger, A. flavus,* and *A. terreus* have been reported as the significant species isolated in IA. Our designed probe can be specifically and sensitively to the major species of IA (*A. fumigatus, A. niger, A. flavus,* and *A. terreus*), in addition to the filamentous ascomycetes. Also, our work additionally detects to azole-resistant *A. fumigatus* and *A. tubingensis* of which strains have high MIC to azole comparing to other *Aspergillus* species. Taken together, our work could be used as the rapid identification and azole resistance in the diagnostic of IA, compared to other studies. In Discussion part, we discussed it in the revised version (line 297-302 ).**

2. Lines 56-58, Introduction part: -These steps are shared between your method and "identification via the polymerase chain reaction" methods. - What are the merits of your method compared to other molecular methods such as multiplex PCR?

**: As you commented, “molecular identification is limited” should be unclear. We intended to improve the limitations of current time-consuming filamentous fungal identification by sequencing-based methods. Therefore, it is modified as “sequencing-based identification is time-consuming” (line 54-55).**

**Compared to multiplex PCR, our method has the advantages of the probe-based real time PCR, so it can be more sensitive, more specific, faster, and used in a quantitative method. Also, our probe-based method could be a time effective way because it does not need additional time for the sequencing (line 233-235 in the section Discussion).**

3. Line 215, discussion part: In average, how much time was reduced compared to other methods?

**: It usually takes 3-14 days of fungal culture for conidia production (Different culture time for each clinical strains), 2-3 days for PCR & sequencing, and 2-3 days for azole-resistant MIC analysis. Therefore, on average, it takes 7 to 20 days. However, in this study, we increased sensitivity and detected in the early stages of hyphae generation, and we performed probe-based real time PCR that did not perform sequencing. As a result, the time required for diagnosis after culture was reduced to 2 ~ 3 days. In other words, it has been reduced by 3 to 14 days compared to the previous method.**

**This has been modified in the introduction (line 54-57) and discussion (line 294-296).**

Reviewer #2:

The manuscript is relevant for PlosOne but there are some issues. You have a very good results but I would like that you explain more about material and methods used to synthesize the control DNA.

When you explain about the standard curve, never show the efficiency (E). However in M&M is explianed that it has been calcualted.

Line 127: What is the protocol used to synthesized the control DNA?

**: I appreciate that you reviewed our paper in detail.**

**As you commented, our explanation could be insufficient. So we described more in materials and methods (lines 124-131) as below.**

**At first, we aligned each consensus sequence of *benA* in *Aspergillus* species (S1 Fig). We used *benA* sequence of *A. fumigatus* as the template and designed to have each probe sequence (conserved probe of filamentous ascomycetes, *Fumigati*, *Nigri*, *Flavi*, and *Terrei*) (Table 2). And then, the designed DNA was synthesized genetically (Bionics Corporation, Seoul). We cloned it into the pUC57 plasmid, and it was transformed into *Escherichia coli* BL21strain. Plasmid DNA of transformants was extracted, tested for the multiplex, and used as control DNA.**

Line 130: why has the author synthesized a control DNA instead to clon different fragments of DNA as positives controls?

**: If different fragments are used as controls, several control-set should be needed. More controls need more wells. In clinical settings, more control leads to fewer test samples, especially in quantitative analysis. Therefore we designed a synthesized control including all probes (5 probes). We describe it at the section of Material & Method and Discussion (line 124-131, line 279-281).**

Results: Line 167: delete a "were not"

**: I thank you for reviewing in detail. We had been corrected it (line 177-178).**

Line 188: The author should include the Efficiency (%) calculated, as explianed in M&M, to each standard curve.

**: As you commented, we added efficiency (%) in the revised version. The amplification efficiency of each probe was found to be 89.8% ~ 104.8% in gDNA and 90.0% ~ 99.4% in Control DNA. These results are described in the line 192-193 of the results section, and each value is added in Table 4.**

Reviewer #3:

1-DNA extraction and purification
Authors indicated: “ In this study, we optimized methods to extract mold DNA. First, freeze-thawing and bead-beating methods were used for mechanical lysis, and different lysis and protein removal solutions were used for the conidia and hyphae (hyphae, Trizol solution and isopropanol; conidia, Epicentre’s lysis buffer and protein precipitation solution). Different lysis buffers were used because Trizol reagent”.
Please, for this issue referr to previous validations of fungal DNA extraction, where it was shown that columns and magnetic beads allowed collecting DNA and separate PCR inhibitors, but detection rates could not be related to DNA-avidity of beads or to elution but to the lack of proteolysis.
Please refer to:
-Goldschmidt P, Degorge S, Merabet L, Chaumeil C. Enzymatic treatment of specimens before DNA extraction directly influences molecular detection of infectious agents. PLoS One. 2014 Jun 17;9(6):e94886. doi:10.1371/journal.pone.0094886.)
-Hsu M, Chen K, Lo H, Chen Y, Liao M, et al. (2003) Species identification of medically important fungi by use of realtime LightCycler PCR. J. Med. Microbiol 52: 1071–1076.

**: I appreciate that you reviewed our paper in detail.**

**We reviewed the two papers which you referred to. Both papers showed better removal of PCR inhibitors using lyticase and protease K. In these papers you mentioned, only a few strains of filamentous fungus (mold) were tested. We did lysis of clinical and environmental mold using enzyme and Qiagen purification kit. However, their purity was not good for quantitative analysis, and many variables among species and strains. So, we tried to look for the efficient lysis and complete removal of protein and PCR inhibitors, and then it has been tested in hundreds of mold strains.**

**We used a bead beating lysis method using Trizol reagent (invitrogen) in the case of hyphae and lysis solution of MasterPure ™ Yeast DNA Purification Kit (Epicentre) in conidias. In addition, we performed purification using phenol/chloroform or precipitation buffer (kit), and then they were additionally purified by the column kit (Qiagen). In tested mold strains, these methods performed complete physical lysis and a fungal DNA purification with the high purity for the quantitative analysis. Trizol agent is more economical and effective in hyphae, but it showed the incomplete lysis in conidia of some strain. The lysis solution of the kit showed the complete lysis and removal of PCR inhibitor in both hyphae and conidia.**

**Some researchers suggested bead beating could be more efficient at lysing fungus than enzymatic lysis (EAPCRI method for Aspergillus DNA from whole blood) (Rosemary A Barnes, P Lewis White, C Oliver Morton, Thomas R Rogers, Mario Cruciani, Juergen Loeffler, J Peter Donnelly, Diagnosis of aspergillosis by PCR: Clinical considerations and technical tips. Medical Mycology 2018 56(suppl_1):60-72.** [**https://doi.org/10.1093/mmy/myx091**](https://doi.org/10.1093/mmy/myx091)**).**

**We discussed it in the Discussion part (line 241-257)**

2- As for potential clinical use it should be stressed that the yields of DNA extraction and the PCR inhibitors must be monitored (i.e. by adding internal controls to each sample). results of the systematic validation of this issue were not found in the manuscript.
**: Actually, we monitored of PCR inhibitors using SPUD plasmid DNA (insert in pUC57 vector) as an internal control. It is a routine task and I did not write it in the paper. However, I regret that it was not mentioned in the first version of our paper. We add the monitoring results in the revised version.**

**We performed an experiment to determine the presence of a PCR inhibitor. In the real time PCR process, the experiment was conducted by spiking SPUD DNA with a 1.3 X 10^5^ copy number. When the difference of Cq value was <1 compared to the SPUD DNA diluted in PBS, it was determined that there was no PCR inhibitor. Also, we checked the yields of DNA extraction by monitoring the mass of genomic DNA. When fungal DNA was extracted in ~10^8^ of conidia, and the amount is >3.5μg (usually 4-5 μg) measured by Quant-iT™ PicoGreen™ dsDNA Reagent (Invitrogen), we used it in our study (the mass of one genome copy of Aspergillus was calculated to around 40 fg). We described it in the section of Material and Method and Result. (S3 Fig, line 159-166, 226-228).**

3- Resutls of LOQ and LOD were 40 fg and 400 fg, respectively. Here, in addition to the quantitative analysis expressed as DNA mass (pg, fg, etc), the sensitivity of the test for each sample should be also related to the number of colony forming units.
For further clinical use, the detection limits should be be also compared with fresh titrated fungal suspensions seeded into the haemoculture system (inoculums containing serial diluted CFU/bottle).
Please, refer to:
-Obara H, Aikawa N, Hasegawa N, Hori S, Ikeda Y, et al. (2011) The role of a real-time PCR technology for rapid detection and identification of bacterial and fungal pathogens in whole-blood samples. J Infect Chemother 3: 327–33.
-Goldschmidt P, Degorge S, Che Sarria P, Benallaoua D, Semoun O, Borderie V, Laroche L, Chaumeil C. New strategy for rapid diagnosis and characterization of fungal infections: the example of corneal scrapings. PLoS One. 2012;7(7):e37660.)

**: As you mentioned, it could be a better way to describe the sensitivity related to the number of colony-forming units (CFU). However, CFU of the filamentous fungus (mold) is difficult to be counted due to the existence of hyphae and conidia (live/dead), the different growth rates of each conidia, error rate of hemocytometer counting, and so on. Therefore, the quantitative analysis from CFU of mold is very variable, and the validation should be difficult. We decided to validate our method by DNA mass related to fungal genome copy. Although the graphs and graphs are the mass of DNA (pg, fg), the genome copies number was obtained using the genome size and molar concentration, and we described in the text (Fig 1, Line 193-197, 220-224).**

**Therefore, we statistically described multiplex real time PCR results of LOQ and LOD in this paper, not the method of fungal DNA extraction. As you mentioned, we will approach the LOQ and LOD both in fungal DNA extraction and multiplex real time PCR from clinical specimens for clinical use in further study.**

**We will discuss it in the section of Discussion (line 304-305).**

4- Results of specificity of the different set of primers with no filamentous fungi were not found.
-Marr KA, Carter R, Crippa F, Wald A, Corey L (2002) Epidemiology and outcome of mould infections in hematopoietic stem cell transplant recipients. Clin Infect Dis 34: 909–17.
-Horvath L, George B, Murray C, Harrison L, Hospenthal D (2004) Direct comparison of the BACTEC 9240 and BacT/ALERT 3D automated blood culture systems for Candida growth detection. J Clin Microbiol 42: 115–8.

**: We designed a probe able to detect filamentous ascomycetes. Besides, non-ascomycetes and non-filamentous ascomycetes were used in the specificity of the test, and these results were described (Line 171-173). In one of non-filamentous ascomycetes (No. 39, Table S1), and 8 of non-ascomycetes molds (No. 40-47, Table S1), there was no cross-reaction in all set of probes. In the designed real time PCR in this study, the fungal DNA of filamentous ascomycetes (No. 1-38, Table S1) was amplified specifically. (Table 3, Table S1)**
